# Hypoxia‐Driven Regulation of Osteogenic Differentiation in Human Periosteal Stem Cells via the HIF‐1α/miR‐129‐5p/ *BMP2* Axis

**DOI:** 10.1111/jcmm.70703

**Published:** 2025-07-08

**Authors:** Jiajia Lu, Xinyu Wang, Nan Lu, Aimin Chen, Lianbo Xiao

**Affiliations:** ^1^ Department of Orthopedic Trauma, Shanghai Fourth People's Hospital, School of Medicine Tongji University Shanghai People's Republic of China; ^2^ Department of Orthopedic Guanghua Hospital Affiliated to Shanghai University of Traditional Chinese Medicine Shanghai People's Republic of China; ^3^ Department of Orthopedic Trauma, Shanghai Changzheng Hospital Shanghai People's Republic of China

**Keywords:** *BMP2*, *HIF‐1α*, human periosteal stem cells, hypoxia, *miR‐129‐5p*, osteogenic differentiation

## Abstract

This study aims to elucidate the regulatory mechanisms of osteogenic differentiation in human periosteal stem cells (hPSCs) under hypoxia, focusing on the *HIF‐1α/miR‐129‐5p/BMP2* axis. RNA‐seq identified miRNAs involved in hypoxia‐induced differentiation, and key molecules were validated at molecular and cellular levels. Hypoxia significantly enhanced osteogenic differentiation, as demonstrated by increased mineralization and upregulated osteogenic markers (*BMP2*, *RUNX2*, and *Osterix*). *miR‐129‐5p* was downregulated under hypoxia, and its overexpression suppressed *BMP2* expression, indirectly affecting *HIF‐1α* levels and weakening osteogenic differentiation. *HIF‐1α* was identified as an upstream regulator of these miRNA changes. These findings provide new insights into the role of hypoxia in bone regeneration and offer potential strategies for bone repair and stem cell therapy.

## 1 Introduction

The periosteum is a connective tissue membrane covering the bone surface, rich in stem and progenitor cells, and plays a crucial role in bone injury and repair [1]. However, the specific cells driving periosteal osteogenesis and chondrogenesis remain unclear. The impaired healing in bone injuries lacking periosteum suggests the presence of a unique stem cell population [2]. Debnath et al. first identified periosteal stem cells (PSCs) and isolated them from mouse long bones and tibiae, demonstrating their multipotency to differentiate into bone, cartilage, and adipose tissue [3]. As the primary source of osteoblasts (OBs) and chondrocytes in the periosteum, PSCs are essential for bone healing and provide new insights into bone repair and maintenance [4, 5]. However, fracture healing occurs in a hypoxic environment, and the impact of hypoxia on PSC osteogenic differentiation remains largely unexplored [5].

Hypoxia is a key microenvironmental factor in fracture healing, regulating cell function by inducing hypoxia‐inducible factors (HIFs) [6, 7, 8]. *HIF‐1α*, the primary regulator, influences cell proliferation, migration, and differentiation by modulating various downstream signalling pathways. Specifically, *HIF‐1α* promotes PSC osteogenic differentiation by regulating gene expression and metabolic pathways [9, 10]. Studies have shown that hypoxia enhances osteoblast activity and improves osteogenic differentiation efficiency, providing crucial theoretical insights into bone formation under hypoxic conditions [11, 12].

Non‐coding RNAs play a key role in osteoblast development, with miRNAs being major regulators that modulate osteogenic gene expression through epigenetic mechanisms. Dysregulated miRNA expression is crucial for stem cell differentiation into osteoblasts and chondrocytes and is implicated in bone diseases [13]. Studies have shown that *miR‐129‐5p* promotes the osteogenic differentiation of periodontal stem cells and PSCs [2, 14] and is negatively correlated with osteogenic markers in femoral tissues of C57BL/6 mice at different ages [15]. However, the precise regulatory mechanisms of *miR‐129‐5p* in osteoblast development remain unclear.

Bone morphogenetic protein 2 (*BMP2*), a member of the transforming growth factor‐β (TGF‐β) family, plays a crucial role in stem cell osteogenic differentiation and bone regeneration [16, 17]. Osteogenesis imperfecta is primarily caused by mutations in COL1A1 or COL1A2, and *BMP2* promotes osteoblast‐mediated collagen synthesis, particularly type I collagen (COL1), the main component of the bone matrix, which is essential for bone structure and strength [18]. However, whether *BMP2* is involved in the osteogenic differentiation of PSCs remains unclear.

This study provides the first investigation into the effects of hypoxic stress on PSCs. By modulating miR‐129‐5p and BMP2 expression in PSCs, the regulatory role of the *miR‐129‐5p*/*BMP2* axis in hypoxia‐induced osteogenic differentiation was examined. These findings offer strong scientific evidence and novel mechanistic insights into hypoxia‐mediated regulation of PSC osteogenic development.

## 2 Materials and Methods

### 2.1 Establishing Mouse Models Under Hypoxic and Normoxic Conditions

A total of 20 male C57BL/6N mice (6–8 weeks old, 20–25 g) were purchased from Beijing Vital River Laboratory Animal Technology Co. Ltd. (Catalogue No. 213, Beijing, China) and housed in a specific pathogen‐free (SPF) animal facility under controlled conditions (humidity: 60%–65%, temperature: 22°C–25°C). After 1 week of acclimation, the mice were randomly assigned to a normoxia group and a hypoxia group (*n* = 10 per group). The normoxia group was maintained under standard conditions, while the hypoxia group was exposed to a hypobaric hypoxic environment for the first 3 days post‐surgery (Table S1), followed by normoxic conditions for the remaining 8 weeks.

Mice were deeply anaesthetised with ether, and a mid‐diaphyseal femoral fracture was induced under sterile conditions using a transverse osteotomy. The fracture was stabilised with an intramedullary stainless steel pin (0.5 mm diameter, Sigma‐Aldrich, USA) to ensure fixation.

At the end of the experiment (8 weeks post‐fracture), mice were anaesthetised with ether and euthanised. The fracture site and surrounding tissues, including the periosteum, were surgically collected under sterile conditions. Periosteal samples were immediately snap‐frozen in liquid nitrogen for subsequent molecular and histological analyses.

### 2.2 X‐Ray Imaging and Analysis

In the 4th and 8th weeks of the experiment, X‐ray imaging was utilised to assess the fractures in the mice. The X‐ray imaging system was the ZEISS Xradia 520 Versa (Zeiss, Germany). Before imaging, the mice were administered proper anaesthesia to ensure they remained still. The X‐ray imaging system was configured with a voltage of 40 kV, a current of 200 μA, and an exposure time of 10 s. Multiple images of the fracture sites of each mouse were captured to ensure a clear depiction of the fracture lines and callus formation. The collected X‐ray images were analysed using Image J software (Image J 1.52, Wayne Rasband, USA) to assess the clarity of the fracture lines and the density of the callus, thereby determining the progress of fracture healing. The impact of a low‐oxygen environment on fracture healing was analysed by comparing the imaging data between the normoxia group and the hypoxia group.

### 2.3 Micro‐CT Imaging and Analysis

In the 4th and 8th weeks of the experiment, Micro‐CT imaging was employed to evaluate the fractures in the mice. The Micro‐CT imaging system used was the SkyScan 1176 (Bruker, Belgium). Prior to imaging, the mice were appropriately anaesthetised to ensure they remained motionless during the imaging process. The Micro‐CT imaging system was set with a voltage of 50 kV, a current of 200 μA, an exposure time of 10 s, and a spatial resolution of 9 μm. Each mouse's fracture site underwent a 360° rotational scan to obtain high‐resolution three‐dimensional images. The collected Micro‐CT images were reconstructed using NRecon software (Bruker, Belgium), and image analysis was performed using CTAn software (Bruker, Belgium) to evaluate the clarity of fracture lines and the density of the callus, determining the progress of fracture healing. The influence of a low‐oxygen environment on fracture healing was investigated by comparing the imaging data between the normoxia group and the hypoxia group.

### 2.4 Pathological Analysis

Morphological changes in periosteal tissue under normoxic and hypoxic conditions were evaluated. Fractured femurs and surrounding periosteal tissues were fixed in 4% paraformaldehyde (PFA), followed by dehydration, clearing, and paraffin embedding. All hard tissue samples underwent decalcification. Tissue sections (5 μm thick) were stained with H&E, Masson's trichrome, and subjected to immunohistochemical analysis. Stained sections were examined under a light microscope (Zeiss, Germany) to assess nuclear and cytoplasmic morphology.

After deparaffinisation, antigen retrieval was performed in citrate buffer (pH 6.0) using microwave heating for 10 min, followed by cooling to room temperature. Endogenous peroxidase activity was blocked by incubating sections in a 3% hydrogen peroxide solution for 30 min. The sections were then incubated overnight at 4°C in blocking solution containing primary antibodies, including *BMP2* (1:200, ab214821, Abcam, USA), *HIF‐1α* (1:100, ab51608, Abcam, USA), *RUNX2* (1:500, ab192256, Abcam, USA), and *Osterix* (1:200, ab209484, Abcam, USA).

The following day, sections were washed with PBS and incubated with HRP‐conjugated secondary antibodies (1:200, Jackson ImmunoResearch, USA) at room temperature for 1 h. DAB staining kit (Thermo Fisher Scientific, USA) was used for colour development until brown signals appeared, with reaction time adjusted based on staining intensity. Haematoxylin counterstaining was performed to highlight cell nuclei. Finally, stained sections were examined under a light microscope (Zeiss, Germany), and image analysis was performed using ImageJ software (ImageJ 1.52, Wayne Rasband, USA) to ensure staining quality and data accuracy and consistency.

### 2.5 Cell Isolation, Culture, and Treatment Protocol

Human periosteal stem cells (hPSCs) were carefully harvested and identified from bone membrane tissue obtained from five volunteer donors, including three males and two females aged between 56 and 70. These tissues were extracted meticulously from bone fracture fragments discarded during orthopaedic surgeries. All procedures involving human participants in this study were conducted by the ethical standards of Shanghai Changzheng Hospital, the 1964 Helsinki Declaration, and its subsequent revisions or similar ethical standards. Prior to participation, all patients and subjects were provided comprehensive information about the study to ensure their full understanding of the nature and implications of the research. Written informed consent was then obtained from each individual.

Upon receipt, the bone membrane specimens were cleaned and then cut into manageable fragments using sterile surgical blades to preserve cell integrity. To extract hPSCs, the tissue fragments underwent a strategic enzymatic digestion process. It involved treating the tissue with Type I collagenase solution (SCR103, Sigma‐Aldrich, USA) and incubating at 37°C for 60 min. The enzyme reaction was rapidly stopped by adding DMEM (21063029, Thermo Fisher, USA) supplemented with 2% fetal bovine serum (FBS). The mixture was then centrifuged to concentrate the cells; the supernatant was removed. For further purification, cells were gently resuspended in DNase I solution (90083, Thermo Fisher, USA) and incubated at 37°C for 5 min to dissolve residual DNA fragments. The samples were systematically filtered through a 70 μm cell strainer to ensure homogeneous single‐cell suspension and eliminate tissue debris. The isolated hPSCs were then cultured in a specialised osteogenic differentiation medium prepared with DMEM, containing 10% FBS, 100 nmol/L dexamethasone, 10 mmol/L β‐glycerophosphate, 0.2 mmol/L L‐ascorbic acid, 100 mg/L penicillin, and 100 U/mL streptomycin, creating an ideal environment for cell growth and differentiation.

Cells were exposed for 3 h in a dedicated hypoxia chamber under controlled conditions of 2% O_2_, 10% CO_2_, and 88% N_2_ to study the effects of hypoxia on hPSCs. Subsequently, hPSCs were carefully divided into different experimental groups based on their redox states: normoxia, hypoxia, hypoxia + NC mimic, *miR‐129‐5p* mimic, and *miR‐129‐5p* inhibitor. These groups laid the foundation for subsequent experimental evaluations and analyses.

### 2.6 Alkaline Phosphatase (ALP) and Alizarin Red S (ARS) Staining Activity Assays

After inducing osteoblast differentiation, cells were washed thrice with phosphate‐buffered saline (PBS) and fixed with 2% paraformaldehyde for 10 min. ALP staining was performed on days 14 and 21 using an ALP staining kit following the manufacturer's instructions, while ARS staining was conducted using pH 4.2 ARS solution for 30 min. Images were captured using a Canon camera and a reflected light microscope (Olympus, Japan, 40× magnification). ImageJ software (ImageJ 1.52, Wayne Rasband, USA) was used to determine the total colony‐forming units of ALP (Cfu‐ALP) and ARS‐positive colonies (Cfu‐ARS).

For quantification, ALP‐stained cells were solubilised using 1% Triton X‐100 solution, and absorbance was measured at 405 nm using a microplate reader (5200110, Thermo Fisher, USA). ARS‐stained cells were dissolved in 10% cetylpyridinium chloride (CPC) solution, and absorbance was measured at 540 nm. The relative ALP and ARS activities were normalised to their corresponding total protein concentrations.

### 2.7 RNA‐Seq Analysis

High‐throughput RNA sequencing was performed to analyse miRNA expression patterns in hPSCs. hPSCs were cultured for 6–8 days under different conditions (hypoxia and normoxia), with some cells induced for osteogenic differentiation under these conditions to assess hypoxia's impact on bone formation. After differentiation, total RNA was extracted using TRIzol (Invitrogen, USA), followed by quantification and purity validation. Following assurance of quality and purity, RNA libraries were constructed using TruSeqTM Small RNA Sample Prep Kits (Illumina, San Diego, USA) per the manufacturer's instructions. The sequencing process was carried out on the Illumina Hiseq2500 platform. Established methods were utilised to remove common RNA families, including rRNA, tRNA, snRNA, snoRNA, adapter dimers, low‐complexity sequences, and repetitive sequences, from the data. Subsequently, Bowtie software was employed to align small RNA‐seq reads with miRbase build 20. Differential expression was determined using normalised deep sequencing counts (RPM, NOISeq), with miRNAs exhibiting expression changes exceeding two‐fold and a *p* value less than 0.05 classified as differentially expressed miRNAs.

### 2.8 Luciferase Reporter Enzyme Analysis

A luciferase reporter enzyme system (6OSE2‐Luc) was developed to accurately investigate the binding sites of BMP2, utilising the PGL3‐basic luciferase vector synthesised by Transheep Bio Co. Ltd. (Shanghai, China). BMP 2 overexpression plasmid (NM_001146038.2), *HIF‐1α* expression plasmid (NM_001154048.2), and Renilla expression plasmid were obtained from Genome Ditech Co. Ltd. (Shanghai, China). Plasmid transfection was conducted using Lipofectamine 3000 transfection reagent following standard procedures. Post‐transfection, the luciferase reporter enzyme activity was assessed using the Dual‐Luciferase Reporter Gene Assay Kit (11405ES, Yeasen, Shanghai, China), strictly following the manufacturer's instructions to ensure accurate and reliable results.

### 2.9 Bioinformatics Prediction

High‐throughput RNA‐seq technology was employed to delve deeper into the miRNA expression patterns in hPSCs. Initially, total RNA from hPSCs underwent quantification and purity validation, with the integrity of these total RNAs evaluated using agarose gel electrophoresis. High‐quality total RNA was reverse transcribed into cDNA, and RNA libraries were constructed for sequencing using Illumina's NextSeq 500 platform. The raw image data from sequencing was processed through base calling to generate raw reads. Cutadapt was utilised to remove sequencing adapter sequences and filter out low‐quality sequences, leaving behind “clean reads” to control the quality of raw reads. The sequences were aligned to the human reference genome using Hisat2 software, followed by quantitative gene expression analysis using the R software package to generate a gene expression matrix. Differential expression analysis was performed using the R package “edgeR”, with a threshold of |log2FC| > 1 and *p* < 0.05 for identifying differentially expressed genes. Additionally, key miRNA target gene lists were obtained from the StarBase website, and the Protein–Protein Interaction Network (PPI) of differentially expressed mRNAs was obtained from the STRING website.

### 2.10 Dual‐Luciferase Assay

The synthetic *BMP2* 3'UTR wt was constructed for the dual‐luciferase assay by designing complementary sequence mutation sites of the wild‐type seed sequence and inserting it into the psiCHECK2 vector. HEK‐293T cells (CL‐0005, Wuhan PuNuoSai Life Science Co. Ltd., China) were transfected with both wild‐type and mutant plasmids, along with *miR‐129‐5p* mimic or NC mimic, using Lipofectamine 2000 (11668019, Invitrogen) following the manufacturer's instructions. The cells were then incubated for 24 h, and the luciferase activity of each group was measured using the Promega Dual‐Luciferase Reporter Assay System (Promega, Madison, WI, USA).

### 2.11 RT‐qPCR

Total RNA from mouse periosteal tissues and cells was extracted using TRIzol (15,596,026, Invitrogen, Carlsbad, CA, USA), and the concentration and purity of the extracted total RNA were assessed using a nanodrop2000 micro‐UV spectrophotometer (1011U, nanodrop, USA). mRNA was reverse transcribed to cDNA using the PrimeScript RT reagent Kit (RR047A, Takara, Japan) following the manufacturer's instructions, while miRNA was reverse transcribed using the TaqMan MicroRNA Assays Reverse Transcription Primer (4427975, Applied Biosystems, USA). Real‐time fluorescence qPCR was performed using an ABI7500 qPCR instrument (7500, ABI, USA) with the following reaction conditions: 95°C for 10 min, 40 cycles of 95°C for 10 s, 60°C for 20 s, and 72°C for 34 s. GAPDH was used as an internal control. The relative gene expression to GAPDH was calculated using the 2^−ΔΔCt^ method: △△Ct = △Ct _experimental group_—△Ct _control group_, where △Ct = Ct _(target gene)_—Ct _(internal control)_. Each experiment was performed in triplicate, and the primers were synthesised by TaKaRa company (Table 1).

**TABLE 1:** Primers sequences for the relevant genes.

Gene name	Primer sequence (5′ to 3′)
*MIR‐129‐5*p (human)	F: 5′‐TTTTGCGGTCTGGGCTT‐3’
	R: 5′‐GAACATGTCTGCGTATCTC‐3’
*Alp* (*Alpl*) (mouse)	F: 5′‐ACACCAATGTAGCCAAGAATGTCA‐3’
	R: 5′‐GATTCGGGCAGCGGTTACT‐3’
*Ocn* (*Bglap*) (mouse)	F: 5′‐GGTAGTGAACAGACTCCGGC‐3’
	R: 5′‐GGGCAGCACAGGTCCTAAAT‐3’
*BMP2* (human)	F: 5′‐AGAATAACTTGCGCACCCCA‐3’
	R: 5′‐GGACCGAATGTCCGTTCCTT‐3’
*Bmp2* (mouse)	F: 5’‐TGCTTCTTAGACGGACTGCG‐3
	R: CTGGGGAAGCAGCAACACTA‐3
*HIF‐1α* (human)	F: 5′‐AGAGGTTGAGGGACGGAGAT‐3’
	R: 5′‐TGGCTGCATCTCGAGACTTT‐3’
*Hif‐1α* (mouse)	F: 5′‐CTGGTGGCTCAGCAGTCTATT‐3’
	R: 5′‐ TGCTGAGGAGCTGTGAATGT‐3’
*RUNX2* (human)	F: 5′‐CGCCTCACAAACAACCACAG‐3’
	R: 5′‐TCACTGTGCTGAAGAGGCTG‐3’
*Ruxx2* (mouse)	F: 5′‐GGGGCAGTCATAACTGGGTT‐3’
	R: 5′‐GCGTGGGAACAGGTCACTTA‐3’
*OSTERIX* (human)	F: 5′‐TGAGTGCCTACTCTGTGCAA‐3’
	R: 5′‐GTGGATGCACCACCAGATCA‐3’
*Osterix* (mouse)	F: 5′‐ACCAGAAGCGACCACTTGAG‐3’
	R: 5′‐TTGGCTTCTTCTTCCCCGAC‐3’
*GAPDH* (human)	F: 5′‐CCATGGGGAAGGTGAAGGTC‐3’
	R: 5′‐GCGCCCAATACGACCAAATC‐3’
*Gapdh* (mouse)	F: 5′‐CTCATGGCCTACATGGCCTC‐3’
	R: 5′‐CCCTAGGCCCCTCCTGTTAT‐3’

### 2.12 Western Blot Analysis of Protein Expression in Cells

According to the instructions, total protein from mouse periosteal tissues and cells was extracted using high‐efficiency RIPA lysis buffer (Beyotime, China) containing 1% protease inhibitor and 1% phosphorylase inhibitor. After centrifugation at 15000 rpm for 15 min at 4°C, the supernatant was collected, and the protein concentration was determined using a BCA assay kit (Thermo, USA). The protein samples were mixed with 5× loading buffer (Beyotime, China) at different concentrations, heated, separated by SDS‐PAGE polyacrylamide gel electrophoresis, and then transferred to a PVDF membrane (Millipore, USA). The membrane was blocked with 5% BSA solution at room temperature for 1 h, followed by overnight incubation with primary antibodies: rabbit anti‐BMP2 (ab284387, 1:1000, Abcam, USA), rabbit anti‐*RUNX2* (ab76956, 1:1000, Abcam, USA), rabbit anti‐*Osterix* (sc‐393325, 1:1000, Santa Cruz, USA), rabbit anti‐HIF‐1α (ab308433, 1:1000, Abcam, USA), rabbit anti‐GAPDH (ab9485, 1:1000, Abcam, USA), rabbit anti‐ALP (ab108337, 1:1000, Abcam, USA), and rabbit anti‐OCN (ab93876, 1:1000, Abcam, USA). The membrane was washed with TBST three times for 5 min each, then incubated with an HRP‐conjugated goat anti‐rabbit IgG secondary antibody (ab205718, 1:2000, Abcam, USA) at room temperature for 1.5 h. After incubation, the membrane was washed with TBST three times for 5 min each and then developed using a Pierce ECL substrate (USA). Protein quantification analysis was performed using ImageJ software by calculating the ratio of the grayscale values of each protein to the internal control GAPDH. Each experiment was repeated three times.

### 2.13 Statistical Analysis

The data were obtained from three independent experiments, each with three repetitions. Values are presented as mean ± standard deviation (SD) for each variable. To compare differences between two groups, an independent samples *t*‐test was utilised, while for multiple group comparisons, one‐way analysis of variance (ANOVA) followed by Tukey's post hoc test was employed.

All statistical analyses were conducted using the SPSS software package (version 22.0, IBM, USA). Data visualisation was generated using GraphPad Prism 9.0. The significance level was set at *p* < 0.05. Data within a 95% confidence interval (CI) were considered reliable estimates of the population parameters. Any *p* value less than 0.05 indicated statistically significant differences between groups.

## 3 Results

### 3.1 Accelerated Bone Fracture Healing in Mice Under Hypoxic Conditions

**FIGURE 1:**
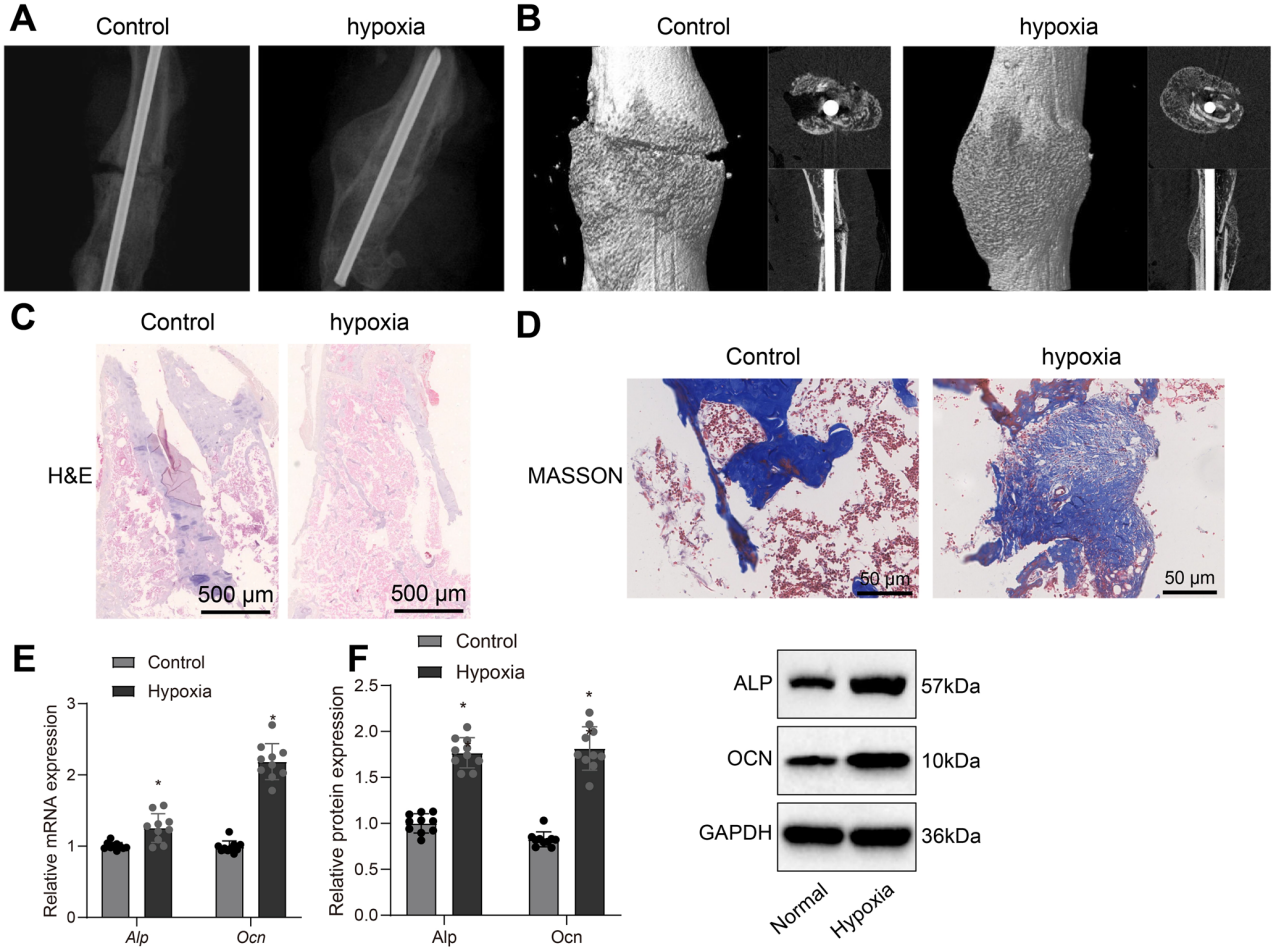
Imaging and histological analysis of mouse fracture sites under hypoxic and normoxic conditions. (A) X‐ray imaging of the normoxia and hypoxia groups at Week 4. (B) Micro‐CT imaging of the normoxia and hypoxia groups at Week 8. (C) H&E staining of the normoxia and hypoxia groups at Week 4, scale bar = 500 μm. (D) Masson's trichrome staining of the normoxia and hypoxia groups at Week 8, scale bar = 500 μm. (E) qPCR analysis of mRNA expression of ALP and osteocalcin (OCN). (F) Western blot analysis of protein expression of ALP and OCN. Each group consisted of 10 mice. Staining results demonstrate the distribution of new bone tissue and collagen fibres. Immunohistochemical analysis was used to assess the expression levels of key proteins, and quantitative analysis was performed using microscopy and ImageJ software. Statistical analysis was conducted using one‐way analysis of variance (ANOVA) and Tukey's post hoc test, with significance set at *p < 0.05, **p < 0.01.

Due to the limitations of human fracture studies, a mouse fracture model was established and maintained under hypoxic and normoxic conditions to investigate the effects of hypoxia on fracture healing. X‐ray imaging and micro‐CT analysis showed that the hypoxia group formed callus more rapidly, with the fracture line significantly blurred by Week 4 and completely disappeared by Week 8, while callus density approached that of normal bone. In contrast, the fracture line remained visible in the normoxia group at Week 8 (Figure 1A,B). HE and Masson's trichrome staining further confirmed that the hypoxia group exhibited more newly formed bone tissue and osteoblasts at Weeks 4 and 8, with a more uniform and abundant distribution of collagen fibres and mineralised bone (*p* < 0.05; Figure 1C,D). Quantitative analysis showed a significant upregulation of bone formation markers ALP and OCN at both mRNA and protein levels under hypoxic conditions (Figure 1E,F).

### 3.2 Hypoxic Stress Significantly Enhances Osteogenic Differentiation Potential of hPSCs

**FIGURE 2:**
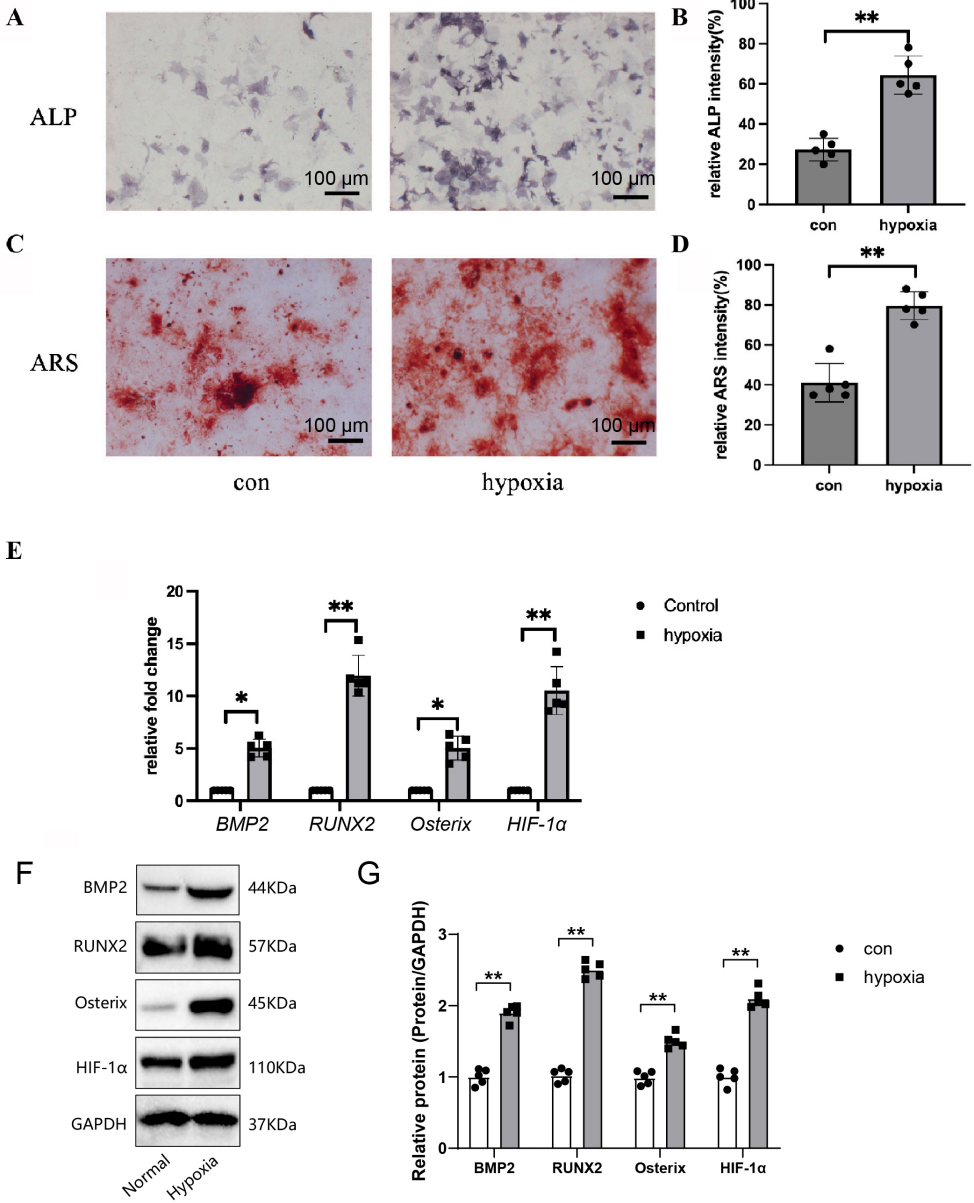
Evaluation of osteogenic differentiation capacity of hPSCs under hypoxic conditions. (A) ALP staining to assess the osteogenic activity of hPSCs under normoxic (con) and hypoxic conditions, scale bar = 100 μm. (B) Quantifying the relative intensity of the ALP‐positive staining area, data presented as mean ± standard error of the mean (SEM), *n* = 5. (C) ARS staining to evaluate the mineralization ability of hPSCs under normoxic and hypoxic conditions, scale bar = 100 μm. (D) Quantification of ARS staining intensity, data shown as mean ± SEM, *n* = 5. (E) qRT‐PCR analysis of mRNA expression levels of osteogenic differentiation‐related factors *BMP2*, *RUNX2*, *Osterix*, and *HIF‐1α* in hPSCs under normoxic and hypoxic conditions, data presented as fold change, mean ± SEM, *n* = 5. (F) Western blot analysis of BMP2, RUNX2, Osterix, and HIF‐1α protein expression levels in hPSCs under normoxic and hypoxic conditions. (G) Quantification of Western blot results, data shown as relative protein expression change, mean ± SEM, *n* = 5. Statistical significance: *p < 0.05, **p < 0.01.

Exploring the effect of hypoxia on the osteogenic differentiation of hPSCs, multiple assays were performed to assess their osteogenic capacity under hypoxic conditions. ALP staining evaluated osteogenic activity, showing a significant increase in ALP‐positive staining area under hypoxia compared to normoxia (*p* < 0.01, Figure 2A,B), indicating enhanced osteogenic activity. Similarly, Alizarin Red S (ARS) staining showed significantly stronger ARS staining in hypoxic conditions (*p* < 0.01, Figure 2C,D), further confirming the promotive effect of hypoxia on hPSCs mineralisation. Expression changes of osteogenic differentiation‐related factors were examined using qRT‐PCR and Western blot analyses (Figure 2E). Hypoxia significantly upregulated *BMP2*, *RUNX2*, *Osterix*, and *HIF‐1α* at the mRNA level (*p* < 0.05, *p* < 0.01). Western blot analysis further confirmed increased protein expression of these factors under hypoxia (*p* < 0.01, Figure 2F,G).

### 3.3 Regulation of *miR‐129‐5p* on Osteogenic Differentiation of hPSCs Under Hypoxic Conditions

**FIGURE 3:**
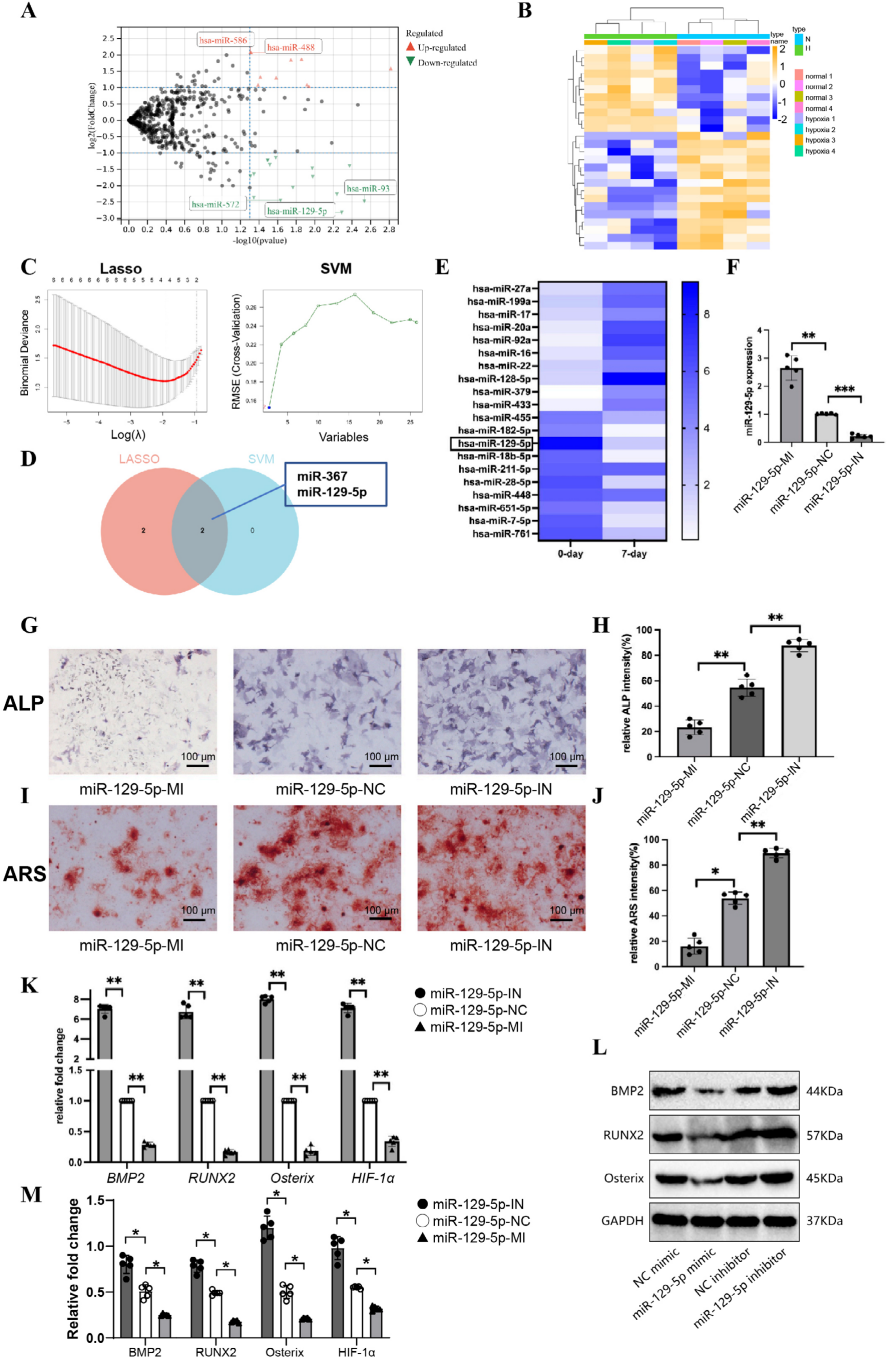
Regulatory role of *miR‐129‐5p* on osteogenic differentiation of hPSCs under hypoxic conditions. (A) RNA‐seq experiment to detect differentially expressed miRNAs in hPSCs under normoxic (con) and hypoxic (hypoxia) conditions. (B) Heatmap illustrating differentially expressed miRNAs under normoxic and hypoxic conditions. (C) Lasso and SVM algorithms were used to identify key miRNAs influencing the osteogenic differentiation of PSCs. (D) Venn diagram displaying common miRNAs selected by Lasso and SVM. (E) qPCR analysis of *miR‐129‐5p* expression levels in hPSCs subjected to normoxic and hypoxic stress, data presented as mean ± standard error of the mean (SEM), *n* = 5. (F) Construction of *miR‐129‐5p* mimic and inhibitor vectors, with NC mimic used as control. (G) ALP staining to detect ALP‐positive staining areas in each group of hPSCs, scale bar = 100 μm. (H) Quantification of the relative intensity of ALP‐positive staining area, data shown as mean ± SEM, *n* = 5. (I) ARS staining to evaluate the mineralization ability of hPSCs in each group, scale bar = 100 μm. (J) Quantification of ARS staining intensity, data presented as mean ± SEM, *n* = 5. (K) qRT‐PCR analysis of the impact of *miR‐129‐5p* upregulation on mRNA expression levels of osteogenic differentiation‐related factors *BMP2*, *RUNX2*, *Osterix*, and *HIF‐1α* in hPSCs, data shown as fold change, mean ± SEM, *n* = 5. (L) Western blot analysis of BMP2, RUNX2, Osterix, and HIF‐1α protein expression levels in hPSCs related to osteogenic differentiation. (M) Quantification of Western blot results, data displayed as relative protein expression change, mean ± SEM, *n* = 5. Statistical significance: *p < 0.05, **p < 0.01, ***p < 0.001.

RNA‐seq analysis revealed significant changes in the miRNA expression profile of hPSCs under hypoxia (Figure 3A,B). Lasso and SVM algorithms further identified key miRNAs, with miR‐367 and *miR‐129‐5p* showing significant alterations under hypoxic conditions (Figure 3C,D). Previous studies have linked *miR‐129‐5p* to osteogenic differentiation in mesenchymal stem cells and renal interstitial fibroblasts [19, 20], leading to its selection for further investigation.

qPCR validation confirmed a significant downregulation of *miR‐129‐5p* under hypoxia (Figure 3E,F). To explore its role in osteogenic differentiation, *miR‐129‐5p* mimic and inhibitor vectors were constructed, with NC mimic as the control.

ALP staining demonstrated that *miR‐129‐5p* overexpression significantly suppressed ALP activity (*p* < 0.01, Figure 3G,H). Similarly, ARS staining revealed that *miR‐129‐5p* overexpression significantly reduced mineralisation (*p* < 0.05, *p* < 0.01, Figure 3I,J).

qRT‐PCR analysis showed that *miR‐129‐5p* overexpression significantly downregulated the mRNA expression of *BMP2*, *RUNX2*, *Osterix*, and *HIF‐1α* (*p* < 0.01, Figure 3K). Western blot results further confirmed a significant reduction in the protein expression levels of these osteogenic factors (*p* < 0.05, Figure 3L,M).

### 3.4 *miR‐129‐5p* Regulates the Expression of *BMP2*

**FIGURE 4:**
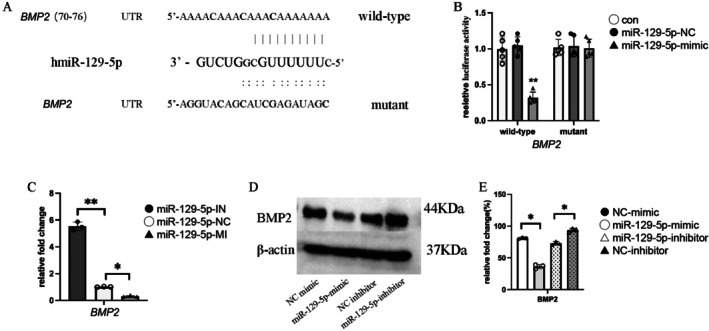
*miR‐129‐5p* regulates *BMP2* expression. (A) Bioinformatics prediction reveals the binding site of *miR‐129‐5p* with *BMP2*. (B) Dual‐luciferase reporter assay validates the regulatory relationship between *miR‐129‐5p* and *BMP2*, results presented as relative luciferase activity, data shown as mean ± standard error of the mean (SEM), *n* = 5. (C) qRT‐PCR detects the mRNA expression levels of *BMP2* in IN (inhibition), NC (normal control), and MI (mimic) groups of hPSCs, data displayed as fold change, mean ± SEM, *n* = 5. (D) Western blot analysis of *BMP2* protein expression levels in hPSCs. (E) Quantification of Western blot results, data shown as relative protein expression change, mean ± SEM, *n* = 5. Statistical significance: *p < 0.05, **p < 0.01.

Bioinformatics analysis predicted a complementary binding site for *miR‐129‐5p* in the 3'UTR of *BMP2* (Figure 4A). A dual‐luciferase reporter assay was performed to validate this prediction by constructing reporter plasmids containing either the wild‐type (WT) or mutant (MUT) *BMP2* 3'UTR sequences. Co‐transfection experiments in HEK293T cells showed that compared to the WT *BMP2* 3'UTR, the MUT *BMP2* 3'UTR exhibited no significant change in luciferase activity upon co‐transfection with the *miR‐129‐5p* mimic (Figure 4B), indicating that *miR‐129‐5p* directly targets the *BMP2* 3'UTR and regulates its expression.

qRT‐PCR and Western blot analyses further confirmed the regulatory effect of *miR‐129‐5p* on *BMP2* expression. In hPSCs, *miR‐129‐5p* overexpression (*miR‐129‐5p*‐MI) significantly decreased *BMP2* mRNA levels, while *miR‐129‐5p* inhibition (*miR‐129‐5p*‐IN) significantly increased *BMP2* mRNA expression (*p* < 0.05, *p* < 0.01, Figure 4C). Western blot analysis showed that *miR‐129‐5p* overexpression markedly reduced BMP2 protein expression, whereas its inhibition led to a significant increase (*p* < 0.05, Figure 4D,E).

### 3.5 *miR‐129‐5p* Regulates hPSCs Osteogenic Differentiation Through *BMP2* Modulation

**FIGURE 5:**
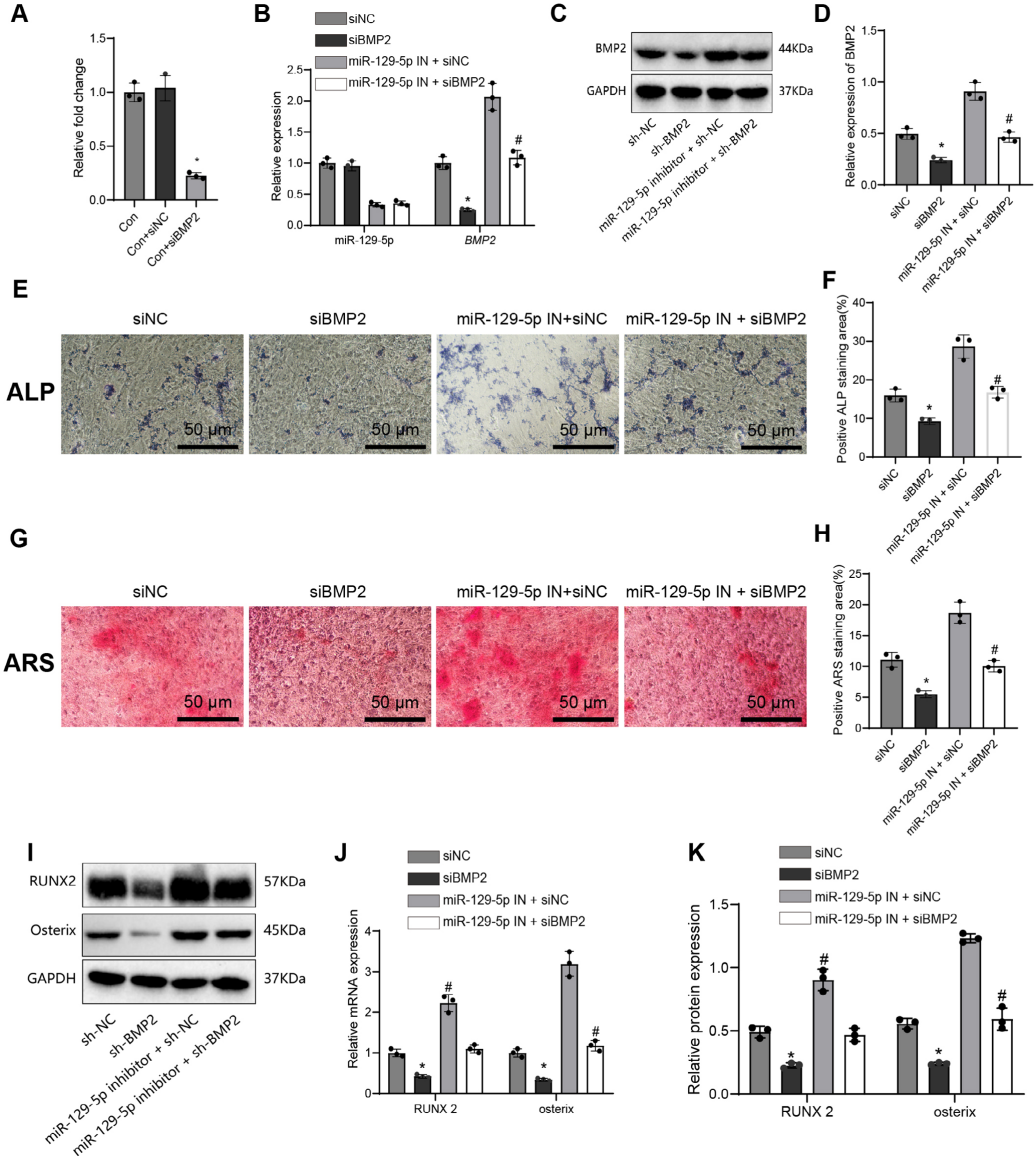
Impact of *miR‐129‐5p* through the *BMP2* gene on osteogenic differentiation of PSCs. (A) qRT‐PCR examines the expression changes of *miR‐129‐5p* and *BMP2* in the control group (con), control with NC group (con+NC), and *BMP2* silenced group (con+si*BMP2*), data presented as fold change, mean ± standard error of the mean (SEM), *n* = 5. (B) qRT‐PCR measures the expression levels of *miR‐129‐5p* and *BMP2* in siNC, si*BMP2*, *miR‐129‐5p* IN + siNC, and *miR‐129‐5p* IN + si*BMP2* groups, data shown as mean ± SEM, *n* = 5. (C) Western blot analyzes the protein expression levels of *BMP2* in siNC, si*BMP2*, *miR‐129‐5p* IN + siNC, and *miR‐129‐5p* IN + si*BMP2* groups. (D) Quantification of Western blot results, data shown as relative protein expression change, mean ± SEM, *n* = 5. (E) ALP staining evaluates ALP‐positive staining areas of hPSCs in siNC, si*BMP2*, *miR‐129‐5p* IN + siNC, and *miR‐129‐5p* IN + si*BMP2* groups, scale bar = 50 μm. (F) Quantifying the relative intensity of the ALP‐positive staining area, data presented as mean ± SEM, *n* = 5. (G) ARS staining examines ARS‐positive staining areas of hPSCs in siNC, si*BMP2*, *miR‐129‐5p* IN + siNC, and *miR‐129‐5p* IN + si*BMP2* groups, scale bar = 50 μm. (H) Quantifying ARS‐positive staining area intensity, data is shown as mean ± SEM, *n* = 5. (I) Western blot assesses protein expression levels of osteogenic differentiation‐related factors *RUNX2 and Osterix* in siNC, si*BMP2*, *miR‐129‐5p* IN + siNC, and *miR‐129‐5p* IN + si*BMP2* groups. (J) Quantifying mRNA expression levels of *RUNX2* and *Osterix*, data displayed as mean ± SEM, *n* = 5. (K) Quantifying protein expression levels of *RUNX2* and *Osterix*, data presented as mean ± SEM, *n* = 5. Statistical significance: *p < 0.05, **p < 0.01, #*p* < 0.001.

To investigate the mechanism by which *miR‐129‐5p* regulates osteogenic differentiation of hPSCs via *BMP2*, siRNA‐mediated *BMP2* silencing was performed, and its effects on *miR‐129‐5p* and *BMP2* expression were evaluated. qRT‐PCR analysis showed that *BMP2* silencing significantly upregulated *miR‐129‐5p* expression while reducing *BMP2* mRNA levels (Figure 5A,B). Western blot analysis further confirmed a significant decrease in BMP2 protein expression (Figure 5C,D). The effect of *BMP2* silencing on osteogenic differentiation of hPSCs was assessed using ALP and ARS staining. Results showed that *BMP2* silencing reduced ALP and ARS staining intensity, whereas *miR‐129‐5p* inhibition partially rescued this suppressive effect (Figure 5E–H). Further, qRT‐PCR and Western blot analyses demonstrated that *BMP2* silencing downregulated the mRNA and protein expression of RUNX2 and Osterix, while *miR‐129‐5p* inhibition partially reversed this suppressive effect (*p* < 0.05, *p* < 0.001; Figure 5I–K).

### 3.6 *miR‐129‐5p* Inhibits *HIF‐1α* Participation in hPSCs Osteogenic Differentiation by Downregulating *BMP2*

**FIGURE 6:**
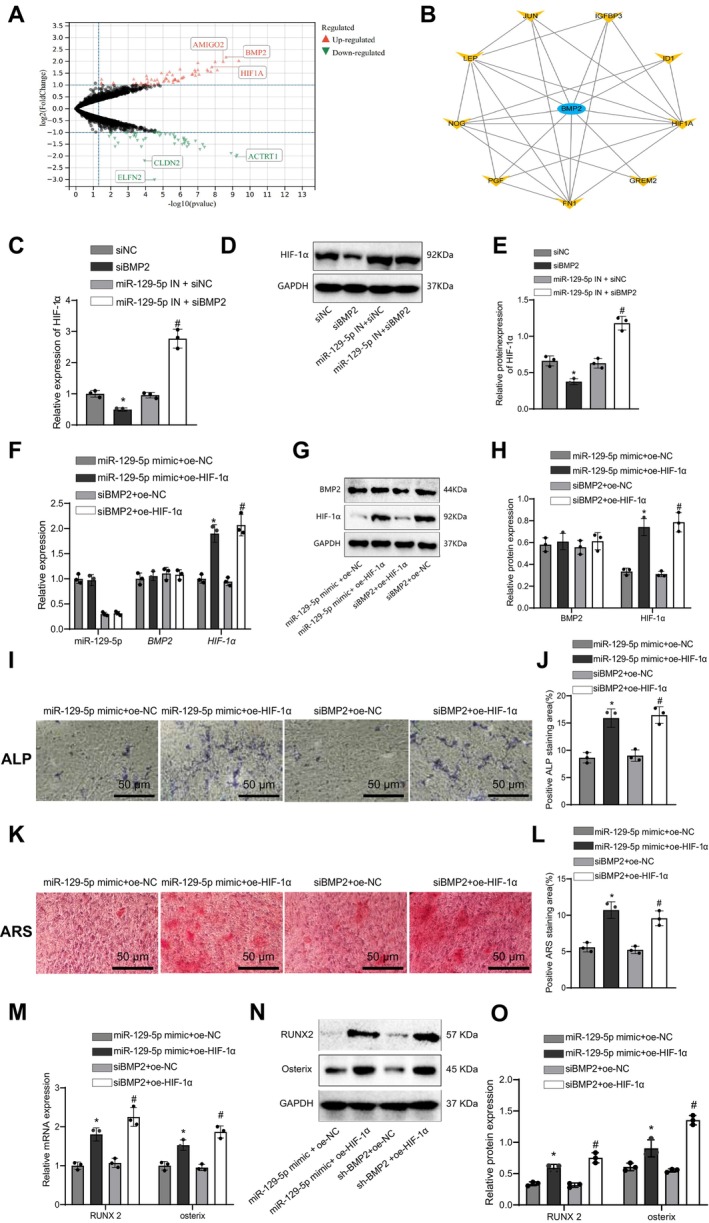
*miR‐129‐5p* regulates hPSCs osteogenic differentiation via the *BMP2*/*HIF‐1α* axis. (A) RNA‐seq reveals significantly differentially expressed genes in hPSCs under hypoxic conditions. (B) PPI network analysis indicates the interaction between *BMP2* and *HIF‐1α*. (C) qRT‐PCR assesses the mRNA expression levels of *HIF‐1α* in siNC, si*BMP2*, *miR‐129‐5p* IN + siNC, and *miR‐129‐5p* IN + si*BMP2* groups, data represented as fold change, mean ± standard error of the mean (SEM), *n* = 5. (D) Western blot examines the protein expression levels of *HIF‐1α* in each group. (E) Quantification of Western blot results displays the relative protein expression changes, mean ± SEM, *n* = 5. (F) qRT‐PCR measures the mRNA expression levels of *miR‐129‐5p*, *BMP2*, and *HIF‐1α* in each group, data shown as mean ± SEM, *n* = 5. (G) Western blot assesses each group's protein expression levels of *BMP2* and *HIF‐1α*. (H) Quantification of Western blot results reveals the relative protein expression changes, mean ± SEM, *n* = 5. (I) ALP staining detects the ALP‐positive staining area of hPSCs in each group, scale bar = 50 μm. (J) Quantifying the relative intensity of the ALP‐positive staining area, data presented as mean ± SEM, *n* = 5. (K) ARS staining evaluates the ARS‐positive staining area of hPSCs in each group, scale bar = 50 μm. (L) Quantification of ARS‐positive staining area intensity, data represented as mean ± SEM, *n* = 5. (M) qRT‐PCR analyzes each group's mRNA expression levels of osteogenic differentiation‐related factors *RUNX2* and *Osterix*. (N) Western blot examines each group's protein expression levels of osteogenic differentiation‐related factors *RUNX2 and Osterix*. (O) Quantification of Western blot results displays the relative protein expression changes, mean ± SEM, *n* = 5. Statistical significance*p < 0.05 and **p < 0.01 when compared to *miR‐129‐5p* mimic + oe‐NC group, #*p* < 0.05 when compared to si*BMP2* + oe‐NC group.

RNA‐seq and bioinformatics analysis identified significantly differentially expressed genes in hPSCs under hypoxia, including *BMP2* and *HIF‐1α*(Figure 6A). Protein interaction network analysis further revealed a strong interaction between *BMP2* and *HIF‐1α* (Figure 6B). qRT‐PCR and Western blot analyses showed that BMP2 silencing significantly downregulated *HIF‐1α* mRNA and protein expression, while *miR‐129‐5p* inhibition partially rescued this effect (Figure 6C–E). In hPSCs, co‐overexpression of *HIF‐1α* and *miR‐129‐5p*/*BMP2* silencing resulted in lower *HIF‐1α* mRNA and protein expression levels compared to the *HIF‐1α* overexpression group alone (Figure 6F–H).

ALP and ARS staining assays demonstrated that *HIF‐1α* overexpression significantly enhanced ALP and ARS staining intensity, while this effect was partially suppressed under *BMP2* silencing and *miR‐129‐5p* overexpression (*p* < 0.05, *p* < 0.05, Figure 6I–L). qRT‐PCR and Western blot analyses further confirmed that *HIF‐1α* overexpression upregulated *RUNX2* and *Osterix* expression, while this effect was also partially suppressed under *BMP2* silencing and *miR‐129‐5p* overexpression (*p* < 0.05, *p* < 0.05, Figure 6M–O).

## 4 Discussion

PSCs exhibit high differentiation potential [21]. Bone defects activate PSCs through multiple signalling pathways, promoting cell migration, proliferation, and differentiation into OBs and chondrocytes at the injury site [3, 22]. However, the mechanisms driving PSC osteogenic differentiation remain unclear. This study demonstrated that hypoxia enhances PSC osteogenesis by mimicking the hypoxic microenvironment during fracture healing. Moreover, the *miR‐129‐5p*/*BMP2* axis plays a key role in hypoxia‐induced PSC osteogenic differentiation.

HIFs are critical regulators in the fracture healing process, responding to local hypoxia and stimulating precursor cell differentiation into osteoblasts [23, 24]. Hypoxia has been identified as a major driver of PSC osteogenic differentiation by upregulating HIFs, thereby modulating periosteum‐regulated osteogenic pathways [25, 26]. Similarly, our findings indicate that hypoxia activates osteogenic differentiation in hPSCs and upregulates osteogenic genes such as *BMP2*, leading to enhanced mineralization. These results highlight hypoxia as a critical factor for hPSC osteogenesis, suggesting that oxygen regulation may be a feasible strategy for inducing osteogenic differentiation in vitro [23].

Our study revealed that *miR‐129‐5p* expression is downregulated under hypoxia. miRNAs are known as key regulators of osteogenesis through gene expression modulation [27]. *miR‐129‐5p* is involved in bone repair, homeostasis, and osteosarcoma cell proliferation [2, 28, 29]. We found that *miR‐129‐5p* overexpression reduces ALP activity, decreases mineralization, and downregulates osteogenic genes such as *BMP2*, ultimately impairing hPSC differentiation into osteoblasts [28].

**FIGURE 7:**
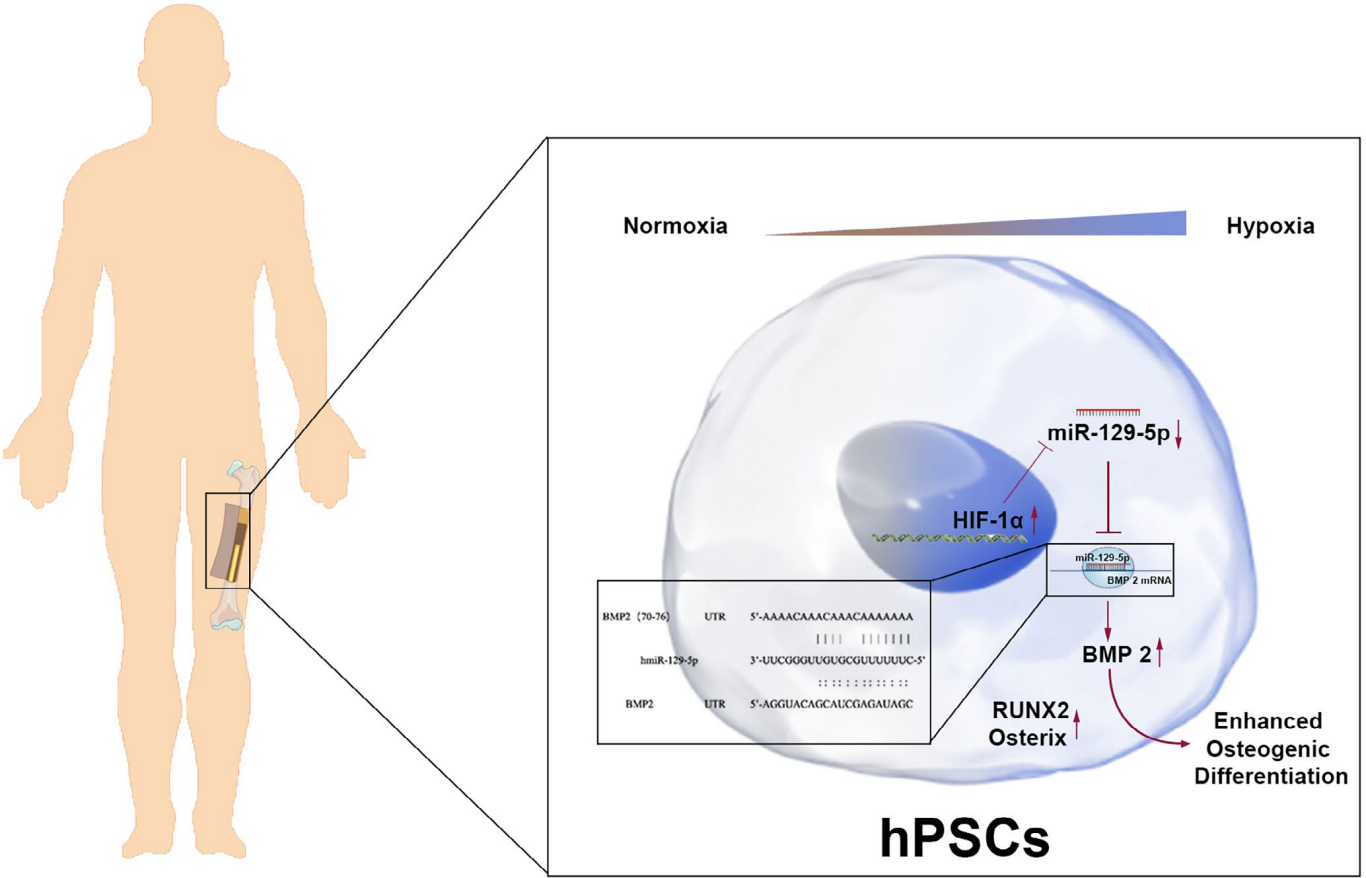
Dual environmental effects of *miR‐129‐5p* on hPSCs osteogenic differentiation.

*BMP2*, a downstream target of *miR‐129‐5p*, is critical in osteogenic differentiation. miRNAs regulate gene expression by interacting with target mRNAs, affecting stability and translation [28]. Bioinformatics analysis identified potential binding sites for *miR‐129‐5p* in *BMP2* mRNA, which was experimentally validated using a dual‐luciferase reporter assay (Figure 7). As a key osteogenic regulator, *BMP2* modulates multiple osteogenic genes and provides the biochemical basis for osteoblast differentiation. BMP2 expression was upregulated to counteract the inhibitory effect of *miR‐129‐5p* overexpression on osteogenesis. These findings suggest that *miR‐129‐5p* suppresses hPSC osteogenic differentiation under hypoxia by targeting *BMP2*.

*HIF‐1α*, a major regulator of hypoxia response, plays a pivotal role in bone regeneration by modulating cell proliferation, migration, and differentiation [30, 31]. However, its specific regulatory role in hPSC osteogenesis remains unclear. Recent studies indicate that *HIF‐1α* enhances stem cell behaviour in hypoxic microenvironments, significantly promoting bone and cartilage regeneration. For instance, *HIF‐1α* activators have been incorporated into biomaterials to mimic hypoxic conditions and enhance bone graft osteogenic potential [32]. Moreover, angiogenesis is closely linked to osteogenesis, and hypoxia has been shown to promote bone regeneration through *HIF‐1α*‐mediated miRNA upregulation, enhancing vascularization. Hypoxia‐preconditioned mesenchymal stem cell‐derived small extracellular vesicles (hypo‐sEVs) significantly promote vascularized bone formation via the miR‐210‐3p/EFNA3/PI3K/AKT pathway, offering a promising strategy for critical‐sized bone defect regeneration [33].

This study elucidates the regulatory role of the *miR‐129‐5p*/*BMP2*/*HIF‐1α* axis in hPSC osteogenic differentiation under hypoxia. *miR‐129‐5p* targets *BMP2*, influencing *HIF‐1α* expression and modulating osteogenic gene expression, ultimately affecting osteogenesis. This study provides the first systematic insight into the role of miRNA regulatory networks in osteogenic differentiation under hypoxia, offering novel perspectives and approaches for future research.

However, this study has some limitations. While hPSC in vitro experiments and a mouse model were utilised, direct validation of hypoxia's effects on human bone tissue was not performed. Although a hypobaric hypoxic environment was used to simulate fracture‐induced hypoxia, its physiological relevance to in vivo fracture healing remains to be fully established.

Future studies should incorporate larger sample sizes, optimised experimental designs, and clinical validation to further confirm these findings. Additionally, further exploration of the specific mechanisms underlying hypoxia‐mediated hPSC osteogenesis could advance stem cell therapy and regenerative medicine, improving treatment strategies for bone injuries and diseases. Future research should also consider alternative regulatory factors and signalling pathways to fully elucidate the complexity of osteogenic differentiation, ultimately enabling precise control of bone regeneration and repair.

## Author Contributions

**Jiajia Lu:** conceptualization (equal), data curation (equal), formal analysis (equal), investigation (equal), methodology (equal), writing – original draft (lead). **Xinyu Wang:** data curation (equal), investigation (equal), software (equal), validation (equal). **Nan Lu:** funding acquisition (equal), investigation (equal), methodology (equal). **Aimin Chen:** conceptualization (lead), project administration (equal), resources (equal), supervision (supporting), writing – review and editing (lead). **Lianbo Xiao:** conceptualization (equal), funding acquisition (lead), project administration (equal), supervision (equal), writing – review and editing (equal).

## Ethics Statement

All procedures involving human participants in this study were conducted by the ethical standards of Shanghai Changzheng Hospital, the 1964 Helsinki Declaration, and its subsequent revisions or similar ethical standards. Prior to participation, all patients and subjects were provided comprehensive information about the study to ensure their full understanding of the nature and implications of the research. Written informed consent was then obtained from each individual.

## Consent

The authors have nothing to report.

## Conflicts of Interest

The authors declare no conflicts of interest.

## Supporting information


**Table S1.** Experimental conditions and parameters.

## Data Availability

The datasets generated and/or analyzed during the current study are not publicly available due to privacy and confidentiality agreements with the participants but are available from the corresponding author on reasonable request.
