# Supplementary material for: Hypoxia‐Driven Regulation of Osteogenic Differentiation in Human Periosteal Stem Cells via the HIF‐1α/miR‐129‐5p/ BMP2 Axis
Source: J Cell Mol Med. 2025 Jul 8;29(13):e70703. doi: 10.1111/jcmm.70703 (PMC12237616; doi:10.1111/jcmm.70703)
Supplement: Supplementary file 1 — Table S1. Experimental conditions and parameters. [file JCMM-29-e70703-s001.docx]

# Table S1 | Experimental Conditions and Parameters.

| Condition | Altitude (m) | Atmospheric Pressure (kPa) | Oxygen Partial Pressure (kPa) | Exposure Duration (Days) |
| --- | --- | --- | --- | --- |
| Normoxia Group | 1500 | 86.3 | 18.0 | 3 |
| Hypoxia Group | 7500 | 35.9 | 8.0 | 3 |
